# T-Cell Responses to *Treponema pallidum* Proteins in Blood and Skin to Advance Syphilis Vaccine Design: Learning From Nature

**DOI:** 10.1093/infdis/jiae246

**Published:** 2024-06-27

**Authors:** Juan C Salazar, Justin D Radolf

**Affiliations:** Departments of Pediatrics; Immunology, University of Connecticut School of Medicine, Farmington; Division of Pediatric Infectious Diseases and Immunology, Connecticut Children's, Hartford; Departments of Pediatrics; Immunology, University of Connecticut School of Medicine, Farmington; Departments of 4 Medicine; Genetics and Genome Sciences; Molecular Biology and Biophysics, University of Connecticut School of Medicine, Farmington

**Keywords:** syphilis, treponema pallidum, vaccine, immune response

**(See the Major Article by Reid et al. on pages 281–92.)**

Syphilis is a chronic, sexually transmitted disease caused by the extremely invasive and immunoevasive spirochetal pathogen *Treponema pallidum* subspecies *pallidum* (*TPA*) [1]. The disease continues to be a major global public health problem, afflicting almost 10 million people annually, including 1.4 million pregnant women [2, 3]. In addition to its potential to cause short- and long-term morbidity in the mother, untreated gestational syphilis can lead to serious adverse outcomes in offspring including stillbirth, prematurity, low birth weight, and neonatal demise [3]. In 2022, a total of 3761 congenital syphilis cases were reported in the United States to the Centers for Disease Control and Prevention via the National Notifiable Diseases Surveillance System, including 231 (6%) stillbirths and 3530 (84%) liveborn infants, with 51 infant deaths [4]. These statistics represent a 31.7% increase in congenital syphilis cases from those reported during 2021, concurrent with a 17.2% increase in rates of primary and secondary syphilis cases among females aged 15–44 years (from 16.3 to 19.1 per 100 000 population). Of additional concern is the worldwide shortage of benzathine penicillin G, the most widely used antibiotic formulation for treatment of syphilis. Collectively, these occurrences underscore the critical need for enhanced public health strategies to curtail syphilis transmission, including development of a safe and effective vaccine with global coverage.

Because *TPA* is an extracellular pathogen, vaccine efficacy is thought to depend upon immunization regimens that elicit antibodies targeting spirochetal surface antigens. Identification of candidate vaccinogens, however, has been complicated and controversial due to the fragility and unusual molecular architecture of the *TPA* outer membrane, most notably its low density of integral membrane proteins that bear little sequence relatedness to well-characterized outer membrane proteins (OMPs) of gram-negative bacteria [5]. Reliance on the outbred rabbit model for evaluating protection following immunization with recombinant proteins further hinders vaccine development [6]. Despite these limitations, the recent characterization of *TPA*'s repertoire of OMPs (the “*TPA* OMPeome”) has provided a road map for target selection [7]. While the ideal candidates have yet to be defined, experts concur that a successful vaccine will require a cocktail of *TPA* surface antigens. There are 2 reasons why cellular immune responses are important for a successful syphilis vaccine. One is to provide strong T-cell help for antibody production against targeted rare OMPs. The other relates to the presumptive mechanism for clearance of *TPA*–macrophage-mediated opsonophagocytosis [8]. Efficient opsonophagocytosis of *TPA* requires macrophages activated by interferon gamma (IFN-γ) [8, 9]. An effective syphilis vaccine, therefore, not only has to target OMPs with antibodies, it also must stimulate cellular responses for B cells in lymph nodes and production of IFN-γ to activate macrophages at sites of infection.

Vaccine development is often based on the “learning from nature” concept (ie, emulating natural protective immunity). Protective immunity in syphilis is not well understood, but it is known that *TPA*-infected rabbits develop resistance to infection [6] and that most humans with untreated syphilis eventually gain control of the disease [1, 6]. Prior studies have screened the *TPA* proteome for antibody reactivity using sera from patients with early syphilis [10]. Presumptive *TPA*-specific memory T cells were identified in the blood and skin of patients with active secondary syphilis, but the antigenic specificities of these cells have not been determined [11]. The impressive study by Reid et al in the current issue of *The Journal of Infectious Diseases* tackles this challenging problem using a novel high-throughput method for protein expression and advanced T-cell expansion and culturing techniques to assess CD4^+^ T-cell responses of syphilis patients to 89 *TPA* proteins. The researchers found responses to 14 proteins—7 of which localize to the outer membrane, 6 to the periplasmic space, and 1 to the cytoplasm. *TPA*-specific reactive CD4^+^ T-helper cells were detectable in blood and skin of syphilis patients at the time of initial presentation and persisted for as long as 10 years following treatment. Significantly, CD4^+^ T-helper cells responded to BamA (β-barrel assembly machinery subunit A-Tp0326), the central component of the molecular machine that inserts new OMPs into the *TPA* outer membrane [7, 12], and 3 members of the FadL family of outer membrane fatty acid importers (Tp0548, Tp0856, and Tp0858) [13]. Of note, BamA, TP0856, and TP0858 already have been identified as vaccine candidates based on antigenicity mapping with sera from syphilis-immune rabbits and opsonophagocytosis experiments using BamA-specific monoclonal and polyclonal antibodies [13, 14]. Indeed, it is tempting to speculate that the robust T-cell responses to these OMPs observed herein explains their immunogenicity for antibodies during syphilitic infection. The strong T-cell response to the periplasmic FlaB1 flagellar protein is in line with an earlier investigation of cellular immunity in *TPA*-infected rabbits [15] and is intriguing as well given a previous report that immunization of rabbits with isolated flagella partially protects against intradermal challenge with *TPA* [16].

While the present work provides important new insights regarding the cellular responses of humans with syphilis, much remains to be done in this nascent area of investigation. Reid et al examined less than 10% of the *TPA* proteome and not the entire OMPeome [17]. Presumably many more potent T-cell immunogens in *TPA* remain to be discovered. Inasmuch as immune cell infiltration of *TPA-*infected tissues is the cause of disease manifestations [1], one should not assume that the detected cutaneous T-cell responses are exclusively protective. And, as the authors note in a surprising but consistent observation from immunohistochemical analyses of T-cells infiltrating syphilitic skin lesions, there is a substantial proportion that are CD8^+^ T cells [18], and they appear to be a principal source of locally produced IFN-γ [19]. Why an extracellular bacterium elicits an exuberant CD8^+^ T-cell response is a major enigma of syphilis immunology.

These groundbreaking studies by the University of Washington group have important implications for the future of syphilis vaccine design. There is now a consensus that syphilis vaccines employing full-length OMPs are fraught with problems. Consequently, the field is turning toward vaccines employing extracellular loops (ECLs) of OMPs, their antibody-accessible regions, displayed on protein scaffolds. The pivot toward ECLs means that T-cell epitopes associated with relevant OMPs will be left behind. Strong B- and T-cell responses in lymph nodes will, therefore, depend upon engrafting scaffolds with potent T-cell epitopes. Vaccine formulations also will need to include epitopes from potent *TPA* T-cell antigens to induce local production of IFN-γ to promote internalization of opsonized spirochetes. Further investigations in humans with syphilis will be needed to identify the optimal T-cell antigens and epitopes to elicit these critical functionally and spatially distinct cellular responses.
